# Six-Month Outcomes from the NEXit Junior Trial of a Text Messaging Smoking Cessation Intervention for High School Students: Randomized Controlled Trial With Bayesian Analysis

**DOI:** 10.2196/29913

**Published:** 2021-10-21

**Authors:** Marcus Bendtsen, Preben Bendtsen, Ulrika Müssener

**Affiliations:** 1 Department of Health, Medicine and Caring Sciences Linköping University Linköping Sweden; 2 Department of Medical Specialist Motala Hospital Motala Sweden

**Keywords:** smoking, cessation, text messaging, high school, randomized controlled trial, intervention, student, young adult, teenager, outcome, Bayesian, Sweden, prevalence, lifestyle, behavior

## Abstract

**Background:**

The prevalence of daily or occasional smoking among high school students in Sweden was approximately 20% in 2019, which is problematic since lifestyle behaviors are established in adolescence and track into adulthood. The Nicotine Exit (NEXit) Junior trial was conducted in response to a lack of evidence for the effects of text message smoking cessation interventions among high school students in Sweden.

**Objective:**

The aim of this study was to estimate the 3- and 6-month effects of a text messaging intervention among high school students in Sweden on smoking cessation outcomes.

**Methods:**

A 2-arm, single-blind randomized controlled trial was employed to estimate the effects of the intervention on smoking cessation in comparison to treatment as usual. Participants were recruited from high schools in Sweden using advertising and promotion by school staff from January 10, 2018, to January 10, 2019. Weekly or daily smokers who were willing to make a quit attempt were eligible for inclusion. Prolonged abstinence and point prevalence of smoking cessation were measured at 3 and 6 months after randomization.

**Results:**

Complete case analysis was possible on 57.9% (310/535) of the participants at 6 months, with no observed statistically significant effect on 5-month prolonged abstinence (odds ratio [OR] 1.27, 95% CI 0.73-2.20; *P*=.39) or 4-week smoking cessation (OR 1.42; 95% CI 0.83-2.46; *P*=.20). Sensitivity analyses using imputation yielded similar findings. Unplanned Bayesian analyses showed that the effects of the intervention were in the anticipated direction. The findings were limited by the risk of bias induced by high attrition (42.1%). The trial recruited high school students in a pragmatic setting and included both weekly and daily smokers; thus, generalization to the target population is more direct compared with findings obtained under more strict study procedures.

**Conclusions:**

Higher than expected attrition rates to follow-up 6 months after randomization led to null hypothesis tests being underpowered; however, unplanned Bayesian analyses found that the effects of the intervention were in the anticipated direction. Future trials of smoking cessation interventions targeting high school students should aim to prepare strategies for increasing retention to mid- and long-term follow-up.

**Trial Registration:**

IRCTN Registry ISRCTN15396225; https://www.isrctn.com/ISRCTN15396225

**International Registered Report Identifier (IRRID):**

RR2-10.1186/s13063-018-3028-2

## Introduction

A steady decline in smoking prevalence has been observed in Sweden over the past decade, with the most recent data indicating that 7% of the general population were daily smokers in 2018 [1]. Although this decline is promising, the prevalence of daily smoking among individuals aged 16 to 29 years was 5% in 2018, and this rate increased to 16% when including occasional smokers. Among Swedish high school students specifically, approximately 5% self-reported being everyday smokers in 2019, and this rate increased to 20% when including occasional smokers [2]. Thus, young individuals in Sweden still start smoking. This is problematic since unhealthy lifestyle behaviors, including smoking, are established in adolescence and track into adulthood [3-5]. Effective smoking cessation interventions that target adolescents are therefore important for the declining trend in smoking prevalence to continue in Sweden.

Mobile phone–based interventions could potentially have far reach among adolescents, as mobile phone ownership in this group is almost universal in Sweden. Of particular interest are text message interventions, as they rely on standard technology that is ubiquitous in all mobile phones, and they have shown promise in other populations. Three meta-analyses have concluded that text messaging interventions have a positive effect on smoking cessation: one reported a summary effect size of 0.25 (95% CI 0.13-0.38) [6], the second meta-analysis reported an overall summary odds ratio (OR) of 1.37 (95% CI 1.25-1.51) of smoking cessation in favor of text messaging interventions [7], and the third analysis similarly found that quit rates were higher among those who had access to text messaging interventions (OR 1.36, 95% CI 1.23-1.51) [8]. Thus, there exists a relatively strong body of evidence for the effects of text messaging interventions. In addition, text messaging interventions may increase access to education and support services that promote smoking cessation [7].

However, only three trials have investigated the effects of smoking cessation text messaging interventions in adolescent populations exclusively [9-11], and only two of these have been conducted in high school student populations [10,11]. Two out of the three were small-scale trials including 179 [11] and 72 [9] participants, respectively, whereas the third was a large-scale cluster randomized trial including 2638 participants [10]. Findings from the large-scale trial suggested that although the number of cigarettes smoked per day was lower among those with access to the intervention, there were no marked differences with respect to smoking abstinence.

In 2018, we conducted the Nicotine Exit Junior (NEXit Junior) trial in response to the lack of evidence for the effects of text message smoking cessation interventions among high school students. We found that a 12-week text message smoking cessation intervention had a positive effect on the 4-week point prevalence of smoking cessation 3 months after randomization among weekly and daily smoking students (OR 1.87, 95% CI 1.12-3.17, *P*=.02) [12]. This report presents the results from the 6-month follow-up of the trial. Analyses prespecified in the study protocol are presented [13], as are unplanned Bayesian analyses of primary outcomes for both the 3- and 6-month follow-ups.

## Methods

### Design

A 2-arm, single-blind, parallel-groups randomized controlled trial was employed to estimate the effects of the NEXit Junior intervention on smoking cessation. The trial was prospectively registered (ISRCTN15396225) and a trial protocol was published in advance of trial recruitment [13]. This report adheres to CONSORT guidelines, and a CONSORT-EHEALTH checklist has been made available with this report. The trial received ethical approval by the Regional Ethical Committee in Linköping, Sweden (Dnr 2017/388-31).

### Participants

Participants were simultaneously recruited from 630 high school units in Sweden. Students were recruited through paper advertising (poster and leaflets) and digital advertising (email, school website, app), and by promotion by school staff (teachers, mentors, school health centers). Recruitment began on January 10, 2018, and ended 1 year later (January 10, 2019).

Students interested in participating in the trial sent a text message to a dedicated telephone number. An automatic response was sent back with a hyperlink to a webpage where information about the trial was presented and students were asked to leave informed consent by pressing a button. Students who consented to participate were taken to a web-based questionnaire including items for both eligibility screening and baseline assessment.

High school students who reported being weekly or daily smokers, and were willing to make a quit attempt, were eligible for the trial. We included weekly smokers for two reasons: (1) this was the criterion for our previous trial of a text messaging smoking intervention among university students [14,15], and (2) weekly smoking in adolescents is cause for concern as it may escalate to daily smoking and long-term dependence. In addition, it was expected that participants have access to a mobile phone throughout the trial period. Individuals reporting not smoking, or doing so on a monthly basis only, were excluded from the trial.

### Interventions

The text message intervention was a 12-week automatic, and unguided, program consisting of a total of 121 messages. During the first 2 weeks of the program, participants received 2 to 4 messages per day, which reduced to 2 messages per day during week 3, 1 message per day during weeks 4 to 7, and on average 5 messages per week during weeks 8 to 12.

The content of the messages was based on a similar intervention targeting university students in Sweden [14,15], which was based on existing evidence-based practice [10,16-22]. The content was further developed and refined for high school students using formative methods and behavior change technique analysis [23-27]. The content of the messages focused on the health risks of smoking and behavior change advice. In particular, participants were asked to make a public declaration about their intention to quit, encouraged to ask for support, taught distraction techniques, given tips on how to cope with cravings, and given advice on how to avoid smoking triggers.

Individuals allocated to the control group were advised that they would not obtain access to the novel intervention and that they instead could use the website of the national quit line [28] or contact their high school’s health service for more help. This was considered treatment as usual at the time of the trial, as this was in general what high school students who wanted help quitting were advised to do.

### Outcomes

At 3 and 6 months after randomization, all participants were sent a text message with a hyperlink to a web-based follow-up questionnaire. Participants were sent two reminders 2 days apart, after which they were called by phone for follow-up (maximum of 10 attempts). The questionnaire contained items for the two primary outcomes: prolonged abstinence and point prevalence of smoking cessation.

Prolonged abstinence, following the Russel standard [29], was defined at the 3-month follow-up as not smoking more than 5 cigarettes in the past 8 weeks. At 6 months, the definition was altered to not smoking more than 5 cigarettes in the past 5 months. This outcome thus measures abstinence from the start of the 12-week smoking cessation program, allowing for a 4-week grace period.

Point prevalence of smoking cessation, a recommended outcome by the Society for Research on Nicotine and Tobacco [30], was defined as not having smoked a single cigarette in the past 4 weeks. This question measures current behavior, and thus was the same at both the 3- and 6-month follow-ups.

### Sample Size

Sample size was determined by assuming that differences in cessation rates between the two groups would be similar to what was observed in a previous study of a text messaging smoking cessation intervention targeting Swedish university students [14,15]. We therefore expected a difference of approximately 10 percentage points between the two groups, which would require 195 individuals in each group to be detected with 80% power at the .05 (two-sided) significance threshold. We assumed that we would have 30% attrition at follow-up, resulting in a required total sample size of 558.

It should be noted that the expected 10 percentage point difference is much higher than what has been synthesized in meta-analyses of text messaging smoking cessation support versus minimal smoking cessation advice. The meta-analyses suggested that the differences may be in the range of 3-4 percentage points [31], which may be a more conservative choice for other trials to adopt. We used a greater difference in consideration of our previous research among university students in Sweden that yielded this difference using a similar intervention and trial design [14,15].

### Randomization

After baseline assessment, eligible students were randomly allocated to the intervention or control group with equal probability. This was done on the backend computer server using Java’s built-in random number generator; thus, the sequence generation was computerized and fully automatic. Allocation was thereby also fully concealed until interventions were assigned.

Research personnel were blind to allocation at the time of randomization; however, participants were informed of which group they were allocated to. Follow-up assessments were completed using online questionnaires by participants on their phones; thus, group allocation was not revealed at the time of follow-up. However, there was a potential risk of group disclosure among those who did not respond to text message prompts and subsequently were called by research personnel to collect follow-up data (see Limitations).

### Statistical Methods

The analyses conformed with the prespecified analyses in the trial protocol [13]. Specifically, logistic regression models were used to compare groups at the different follow-up intervals, adjusting for baseline variables: gender, number of years smoking, average number of cigarettes smoked weekly, severity of dependence (Fagerström Test for Nicotine Dependence), and the use of snuff. Participants were analyzed in the groups to which they were randomized.

Attrition analyses were planned to investigate potential differences between those who did and did not respond to follow-up. A penalized logistic regression model (lasso) was used to identify the baseline characteristics that were potentially predictive of nonresponse. This allowed us to explore departures from the missing completely at random (MCAR) assumption underlying the primary complete case analyses. Sensitivity analyses using multiple imputation with chained equations [32] were performed to assess robustness of the findings (using predictive mean matching, with 500 imputations and 30 iterations).

In addition to the prespecified analyses, unplanned Bayesian analyses were performed. The higher than anticipated attrition rate underpowered the planned null hypothesis tests, and the Bayesian analyses were included to calculate the probability that the intervention had an effect on smoking outcomes. Using a Bayesian approach also removes the emphasis on null hypothesis testing and prespecified significance thresholds, which can be sensitive to single data points and can create counterintuitive results [33-38]. Normal priors with mean 0 and SD 1 were used for all regression coefficients to encode an a priori skepticism of effect. Inference was carried out using the Hamiltonian Monte Carlo procedure (50,000 samples, using the first half as burn-in).

All analyses were performed in R (version 3.6.1); Bayesian inference was performed using the probabilistic programming language Stan (RStan version 2.19.1).

## Results

### Participant Characteristics

**Figure 1:**
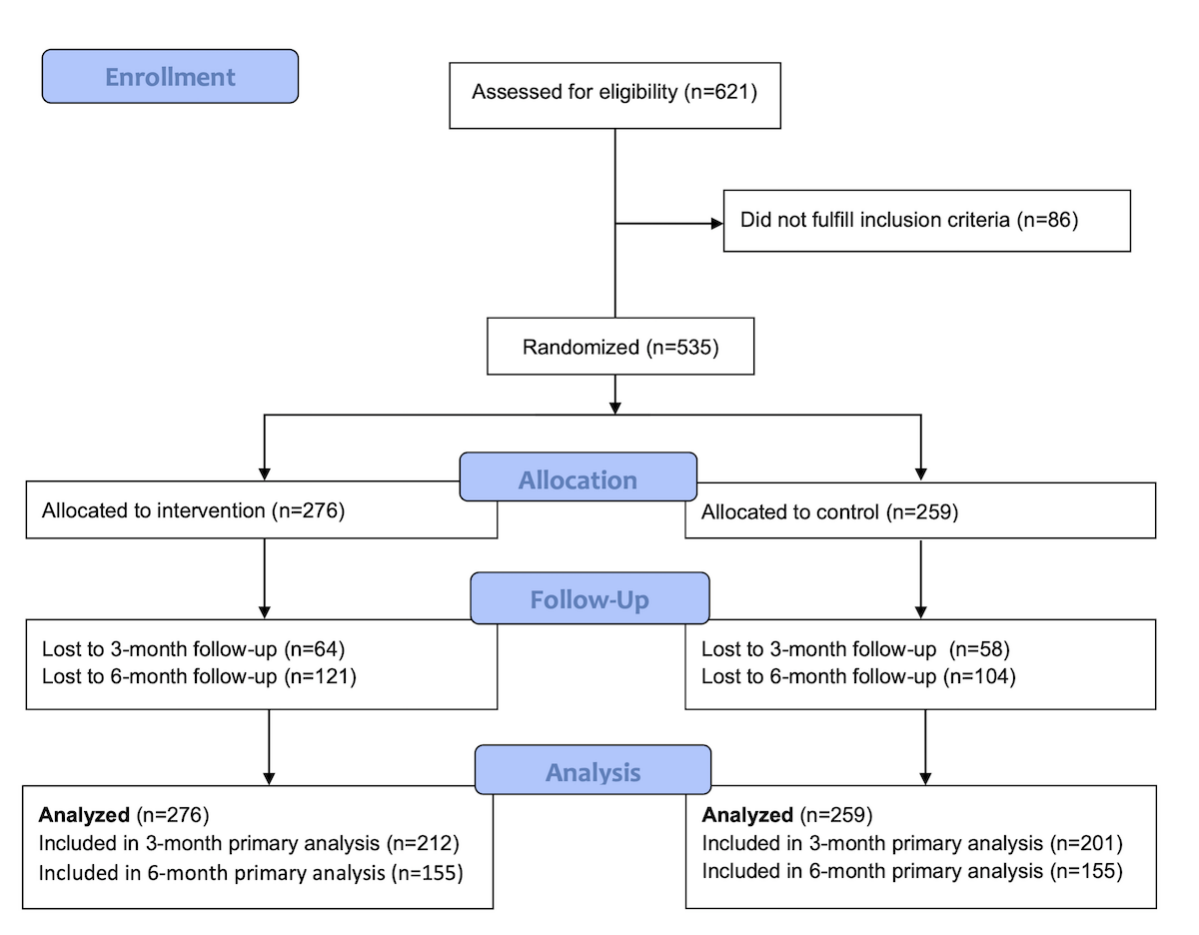
CONSORT flow diagram of the progress through the phases of the Nicotine Exit (NEXit) Junior trial of two groups.

The CONSORT flow diagram in Figure 1 describes the progress of the two groups in the NEXit Junior trial, and Table 1 presents the baseline characteristics of the two groups. A total of 621 high school students were screened for eligibility between January 10, 2018, and January 10, 2019. Among these, 86 students were excluded due to either reporting that they did not smoke on a daily or weekly basis or that they did not want to make a quit attempt, resulting in 535 randomized students. Of those randomized, 276 students were allocated to the intervention group and 259 were allocated to the control group.

**Table 1:** Baseline characteristics of trial participants in the Nicotine Exit (NEXit) Junior trial.

Variables			Intervention (n=276)		Control (n=259)		*P* value
**Gender, n (%)**							.91^a^
	Female	164 (59.4)		162 (62.5)			
	Male	102 (37.0)		90 (34.7)			
	Other	3 (1.1)		2 (0.8)			
	Decline to answer	4 (1.4)		2 (0.8)			
	Do not know	3 (1.1)		3 (1.2)			
Age (years), median (IQR)			17 (16-18)		17 (16-18)		.69^b^
Years smoking, median (IQR)			3 (2-5)		3 (2-5)		.22^b^
Cigarettes smoked per week, median (IQR)			60 (35-84)		70 (42-105)		.06^b^
Regularly using snuff, n (%)			84 (30.4)		74 (28.6)		.71^c^
Fagerström Nicotine Dependence Scale, median (IQR)			4 (2-6)		4 (3-5.5)		.26^b^
Quit attempts, median (IQR)			2 (1-4)		2 (1-4)		.85^b^
Use of nicotine replacement therapies, median (IQR)			0 (0-1)		0 (0-1)		.96^b^
**Cessation counseling experience, n (%)**							.21^c^
	No	250 (90.6)		226 (87.3)			
	Yes, previously	13 (4.7)		22 (8.5)			
	Yes, currently	13 (4.7)		11 (4.2)			
**Recruitment strategy, n (%)**							.46^a^
	Poster	71 (25.7)		80 (30.9)			
	Homepage	49 (17.8)		37 (14.3)			
	Student health center	45 (16.3)		44 (17.0)			
	Staff	34 (12.3)		34 (13.1)			
	School’s mobile app	33 (12.0)		32 (12.4)			
	Friend	21 (7.6)		10 (3.9)			
	Flyer	10 (3.6)		5 (1.9)			
	Email	4 (1.4)		5 (1.9)			
	Other	9 (3.3)		12 (4.6)			

^a^Fisher exact test.

^b^Wilcoxon rank-sum test.

^c^*χ*^2^ test.

### Primary Analysis

Complete case analysis was possible on 57.9% (310/535) of participants at 6 months and on 77.2% (413/535) of participants at 3 months. Sample estimates of prolonged abstinence and smoking cessation (primary outcomes), along with ORs, 95% CIs, and *P* values, are presented in Table 2. Results are presented for both 3- and 6-month outcomes for completeness.

The OR for 4-week point prevalence of smoking cessation, which was statistically significant at the 3-month follow-up, was no longer distinguishable from 1 at the 6-month follow-up at the conventional *P*<.05 threshold; thus, a null finding could not be ruled out. None of the other ORs was statistically significant.

**Table 2:** Primary outcome intention-to-treat analyses with complete cases at 3- and 6-month follow-ups.

Outcomes			Intervention,^a^ n (%)		Control,^b^ n (%)		OR^c^ (95% CI)	*P* value
**3-month outcomes**								
	8-week prolonged abstinence	49 (23.1)		39 (19.4)		1.21 (0.73-2.01)		.46
	4-week smoking cessation	53 (25.0)		31 (15.4)		1.87 (1.12- 3.17)		.02
**6-month outcomes**								
	5-month prolonged abstinence	41 (26.5)		32 (20.6)		1.27 (0.73-2.20		.39
	4-week smoking cessation	44 (28.4)		31 (20.0)		1.42 (0.83-2.46)		.20

^a^n=212 at 3-month follow-up, n=155 at 6-month follow-up.

^b^n=201 at 3-month follow-up, n=155 at 6-month follow-up.

^c^OR: odds ratio based on logistic regression, adjusted for gender, number of years smoking, average number of cigarettes smoked weekly, severity of dependence (Fagerström Test for Nicotine Dependence), and the use of snuff.

### Sensitivity Analysis

We explored potential deviations from the MCAR assumption underlying the primary analyses in Table 1. Penalized logistic regression (lasso) did not reveal any baseline characteristics that were predictive of nonresponse to 6-month outcomes, offering no evidence against the MCAR assumption.

The analyses of 6-month primary outcomes with imputed data found similar effect estimates of the intervention as the complete case analysis for 5-month prolonged abstinence (OR 1.34, 95% CI 0.81-2.20, *P*=.25) and 4-week point prevalence of smoking cessation (OR 1.51, 95% CI 0.93-2.46, *P*=.09). This suggests that the findings in Table 1 are robust to the missing data. Despite no evidence against the MCAR assumption, interpretation of the findings from the imputed values should be made with caution as the rate of attrition (42.1%) was higher than is generally advised when using multiple imputation [32].

### Bayesian Analysis

**Figure 2:**
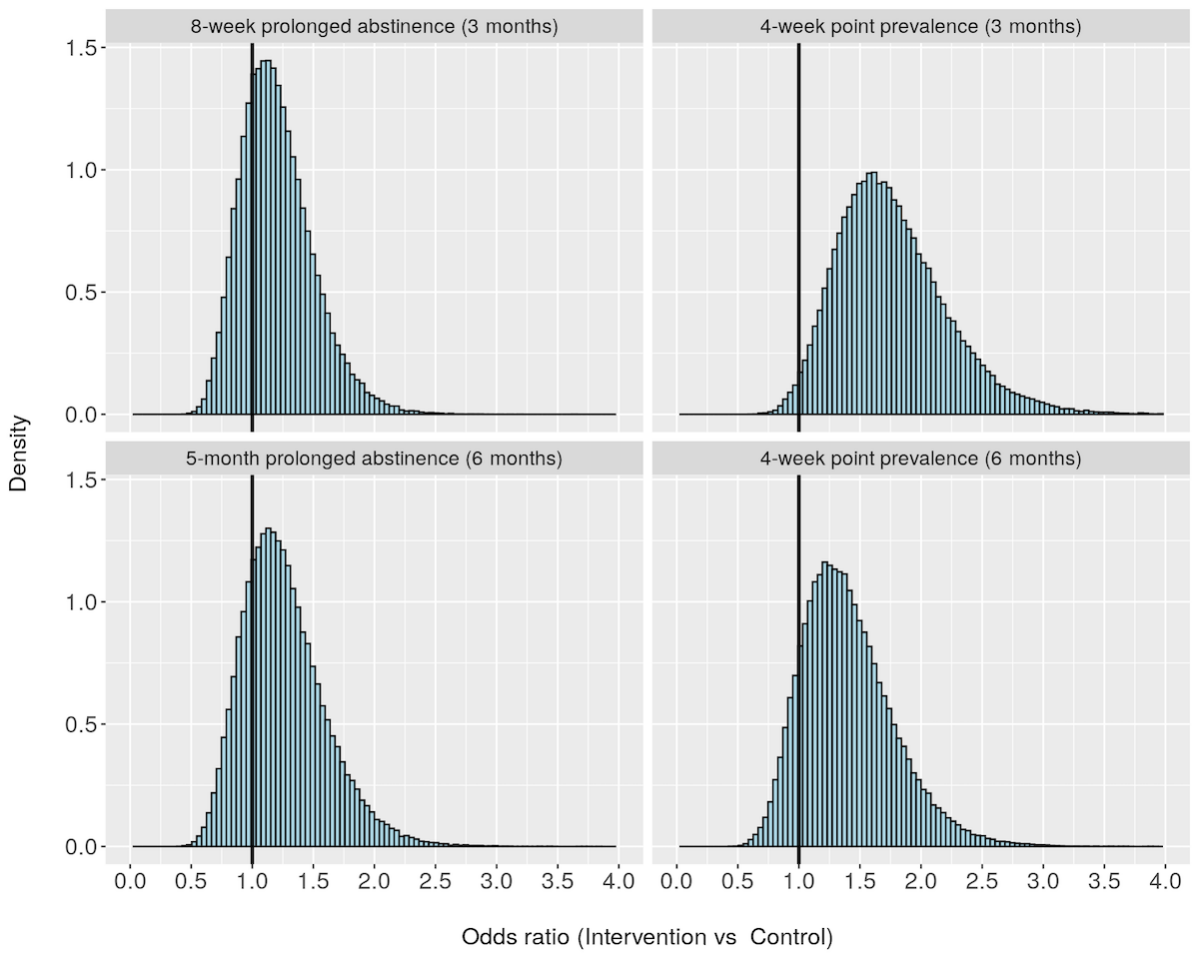
Samples from Hamiltonian Monte Carlo simulations for calculating the odds ratio of intervention vs control.

Figure 2 shows histograms of the samples drawn during the Hamiltonian Monte Carlo simulations. These histograms are representative of the marginal posterior probability of the ORs contrasting the intervention and control groups. The histograms indicate which ORs are more likely than others with respect to the number of samples above or below a specific value. For instance, in the top right histogram, a strong majority of samples lie above 1, indicating a high probability that the intervention had an effect on the 4-week point prevalence of smoking cessation at the 3-month follow-up.

Table 3 presents the probabilities for three different OR thresholds (1, 1.25, and 1.5) for the primary outcomes. The probability that the text message intervention had an effect on prolonged abstinence (ie, OR>1) was 73.8% at 3 months and was 76.9% at 6 months. The probability that the intervention had an effect on 4-week smoking cessation was 98.4% at 3 months and was 87.5% at 6 months. In addition, the probability that the OR was greater than 1.25 for 4-week smoking cessation at 3 months was 89.6% and was 61.7% at 6 months.

**Table 3:** Primary outcome intention-to-treat analyses using Bayesian inference for complete cases at 3- and 6-month follow-ups.

Outcomes		Intervention,^a^ n (%)	Control,^b^ n (%)	Marginal posterior probability (%)		
				OR^c^>1	OR>1.25	OR>1.5
**3-month outcomes**						
	8-week prolonged abstinence	49 (23.1)	39 (19.4)	73.8	38.7	15.0
	4-week smoking cessation	53 (25.0)	31 (15.4)	98.4	89.6	69.8
**6-month outcomes**						
	5-month prolonged abstinence	41 (26.5)	32 (20.6)	76.9	45.6	21.2
	4-week smoking cessation	44 (28.4)	31 (20.0)	87.5	61.7	34.8

^a^n=212 at 3-month follow-up, n=155 at 6-month follow-up.

^b^n=201 at 3-month follow-up, n=155 at 6-month follow-up.

^c^OR: odds ratio obtained by logistic regression, adjusted for gender, number of years smoking, average number of cigarettes smoked weekly, severity of dependence (Fagerström Test for Nicotine Dependence), and the use of snuff.

## Discussion

### Principal Findings

The primary analyses indicated that null findings could not be ruled out when estimating the effect of a 12-week text message intervention on prolonged abstinence and point prevalence of smoking cessation among high school students 6 months after randomization. Sensitivity analyses where missing data were imputed indicated that these findings were robust. Unplanned Bayesian analyses of both 3- and 6-month outcomes indicated that the effects of the intervention were in the anticipated direction.

Among the several studies of smoking cessation text message interventions to a wide range of populations carried out over the past decade, a follow-up period of 6 months is not uncommon. A meta-analysis from 2015 summarized the evidence of nine studies at the 6-month follow-up [8], finding an overall standardized OR of 1.35 (95% CI 1.18-1.49), suggesting that there was a small detectable effect. This is in contrast to our findings, which may be severely affected by attrition and targeted a less explored population; however, this also contrasts with the findings of a large pragmatic randomized trial that did not suffer from severe attrition [39]. The pragmatic trial was performed among both treatment and nontreatment seekers in Australia, with a total of 3550 participants being recruited with high retention (86.5% for the 6-month follow-up); nevertheless, no significant effect of a text message program was found on its own or in combination with a tailored online program. Therefore, although the synthesized evidence does suggest that there are persistent effects, there are certainly some questions remaining regarding the size of these effects and their overall generalizability on health for both subpopulations and the general population.

As mentioned in the Introduction, few trials of smoking cessation text messaging interventions have been conducted targeting adolescent populations exclusively [9-11]. The findings have suggested no marked influence of text messages on smoking abstinence, which is corroborated by our findings in this trial. However, our Bayesian analyses do suggest that effect estimates are in the anticipated direction, even after having taken a skeptical a priori view on the magnitude of the effect. Moreover, although compliance is hard to measure when investigating text messaging interventions, as it is not possible to know with any certainty the degree to which participants read and comply with the messages they receive, during this trial, 21% (n=114) of participants decided that they no longer wished to receive any more messages. This number is difficult to interpret without further data, as some may have been displeased about the support received, some may have just been curious about the trial, while others may have quit smoking and not needed any more support. In any case, we believe that retaining 79% of participants to the end of a 12-week intervention should be considered a success in this population; thus, it is feasible to deliver a smoking cessation intervention via text messages to high school students. The question remains if such an intervention can be designed to also be effective in the long term. Future research should therefore carefully consider if text messaging interventions to this population are worth pursuing. If so, factorial trial designs should be employed to assess multiple components simultaneously [40,41], and Bayesian group sequential designs should be used to inform both successful and futile experiments early on [42-44].

Smoking is considered to be the second leading risk factor for disability adjusted life years globally [45]; thus, effective ways to help individuals quit smoking permanently are needed. Synthesized evidence of smoking cessation interventions targeting adolescents suggest that group counseling may be effective in the long term (risk ratio 1.35, 95% CI 1.03-1.77), whereas individual counseling, pharmacological interventions, and digital interventions did not show long-term effectiveness [4]. However, the quality of the body of evidence is low, as there are several concerns regarding risk of bias and heterogeneity. Although an intervention that encourages smoking cessation and promotes abstinence for a period of time may be considered helpful, we should be careful to offer interventions that may take time away from other smoking cessation activities. It is important to develop interventions that utilize digital platforms as an option for young individuals in Sweden, as they are digital natives and nicotine products can only be bought by people 18 years or older. Moreover, text message interventions targeting university students have been found to be more effective among those without a strong nicotine dependence [46], suggesting that acting early by offering text messaging interventions at the high school level may still be an important public health measure.

### Generalizability

Recruitment to the trial was performed to closely mimic how the intervention could be disseminated to high schools in a full-scale implementation (ie, through print and digital advertisement managed by each school). There was no additional contact with participants throughout the trial period; thus, the trial closely resembles how the intervention would be used in routine care. In addition, inclusion criteria were broad, excluding only those who smoked on a monthly basis or less. The trial may therefore be seen as measuring effectiveness rather than efficacy, which strengthens the external validity of the findings. However, the limitations of this trial should be taken into account when interpreting the findings and the generalizability of the results, especially the high attrition rates.

### Limitations

The most severe limitation of the 6-month findings from the NEXit Junior trial was the risk of bias induced by the high attrition rate (42.1%). Although we found no evidence of systematic missingness, and the findings were robust under imputation, high attrition increases the probability that unobserved factors may predict nonresponse, and thereby bias both the primary and sensitivity analyses. This issue has previously been raised as a concern when studying young adults [47], and future trials on this age group need to implement strategies to retain participants, potentially through incentives.

A second limitation was the lack of blinding of trial participants, which may induce performance bias, an issue prevalent in trials of health interventions as participants are often aware that they signed up for a trial of a particular intervention. Performance bias may be the source of the measured effects rather than the interventions themselves; thus, future trials should prioritize making study information to participants consonant with allocation to intervention and control groups. Additionally, caution should be taken when assessing the present findings since the outcomes were self-reported. This does not necessarily warrant a concern of bias in and of itself; however, in combination with the nonblinding of participants, the risk of performance bias is exacerbated.

Finally, although all processes were automated and participants were treated equally within trial groups, there was a risk of detection bias due to calling nonresponders to collect follow-up data. Despite the fact that the research personnel responsible for the telephone interviews were experienced and instructed not to prompt participants to disclose allocation, some participants are expected to reveal the group to which they were randomized to, and thereby create a risk of bias. However, we judged this risk to be inferior to the increased risk of attrition bias, which would have been induced by not collecting outcome data from those not responding to initial attempts. Furthermore, the analyses revealed no differences in baseline or outcome data between early and late responders, suggesting that the risk of disclosure bias did not lead to any actual bias [12].

These limitations may all influence effect estimates by introducing bias. One way to address these sources of bias post hoc is to consider accounting for them in analyses using causal models [48]. Toward this end, future research should consider estimating the magnitude of effects induced by these sources of bias so that unbiased effects of interventions may be estimated.

### Conclusions

Higher than expected attrition rates to follow-up 6 months after randomization led to null hypothesis tests being underpowered. Although null findings could not be ruled out, unplanned Bayesian analyses suggested that the effects of the intervention were in the anticipated direction both at the 3- and 6-month follow-ups. Future trials of smoking cessation interventions targeting high school students should aim to blind participants to avoid risk of performance bias, and also prepare strategies for increasing retention to mid- and long-term follow-up.

When making policy decisions to implement text messaging interventions for smoking cessation in high schools, consideration should be taken to other alternatives with stronger evidence for long-term effects. However, such decisions should also consider that text messaging interventions are relatively cheap to implement and potentially have great reach among those who may benefit. Moreover, although the effects past the 3-month follow-up period have been difficult to establish due to study limitations, the evidence put forth herein suggests that the intervention was more likely than not to have a positive effect, even in the long run. Considering the health benefits that could come from quitting smoking at such an early age, text messaging interventions could be an important part of a public health campaign in this population.

## Appendix

Multimedia Appendix 1 CONSORT-eHEALTH checklist (V 1.6.1).

## Abbreviations

MCAR: missing completely at random
NEXit: Nicotine Exit
OR: odds ratio

## References

[1] (2018). Nationella folkhälsoenkäten - Hälsa på lika villkor. Folkhälsomyndigheten.

[2] (2019). Skolelevers drogvanor 2019 - CAN rapport 187. Centralförbundet för alkohol- och narkotikaupplysning (C.A.N).

[3] Hall WD, Patton G, Stockings E, Weier M, Lynskey M, Morley KI, Degenhardt L (2016). Why young people's substance use matters for global health. Lancet Psychiatry.

[4] Fanshawe T, Halliwell W, Lindson N, Aveyard P, Livingstone-Banks J, Hartmann-Boyce J (2017). Tobacco cessation interventions for young people. Cochrane Database Syst Rev.

[5] Stockings E, Hall WD, Lynskey M, Morley KI, Reavley N, Strang J, Patton G, Degenhardt L (2016). Prevention, early intervention, harm reduction, and treatment of substance use in young people. Lancet Psychiatry.

[6] Mason M, Ola B, Zaharakis N, Zhang J (2015). Text messaging interventions for adolescent and young adult substance use: a meta-analysis. Prev Sci.

[7] Scott-Sheldon LAJ, Lantini R, Jennings EG, Thind H, Rosen RK, Salmoirago-Blotcher E, Bock BC (2016). Text messaging-based interventions for smoking cessation: a systematic review and meta-analysis. JMIR Mhealth Uhealth.

[8] Spohr SA, Nandy R, Gandhiraj D, Vemulapalli A, Anne S, Walters ST (2015). Efficacy of SMS text message interventions for smoking cessation: a meta-analysis. J Subst Abuse Treat.

[9] Mason MJ, Campbell L, Way T, Keyser-Marcus L, Benotsch E, Mennis J, Zhang J, King L, May J, Stembridge DR (2015). Development and outcomes of a text messaging tobacco cessation intervention with urban adolescents. Subst Abus.

[10] Haug S, Schaub MP, Venzin V, Meyer C, John U (2013). Efficacy of a text message-based smoking cessation intervention for young people: a cluster randomized controlled trial. J Med Internet Res.

[11] Shi H, Jiang X, Yu C, Zhang Y (2016). Use of mobile phone text messaging to deliver an individualized smoking behaviour intervention in Chinese adolescents. J Telemed Telecare.

[12] Müssener U, Linderoth C, Thomas K, Bendtsen M (2020). mHealth smoking cessation intervention among high school students: 3-month primary outcome findings from a randomized controlled trial. PLoS One.

[13] Thomas K, Bendtsen M, Linderoth C, Müssener U (2018). mHealth smoking cessation intervention among high-school pupils (NEXit Junior): study protocol for a randomized controlled trial. Trials.

[14] Müssener U, Bendtsen M, Karlsson N, White IR, McCambridge J, Bendtsen P (2016). Effectiveness of short message service text-based smoking cessation intervention among university students: a randomized clinical trial. JAMA Intern Med.

[15] Müssener U, Bendtsen M, Karlsson N, White IR, McCambridge J, Bendtsen P (2015). SMS-based smoking cessation intervention among university students: study protocol for a randomised controlled trial (NEXit trial). Trials.

[16] Free C, Phillips G, Galli L, Watson L, Felix L, Edwards P, Patel V, Haines A (2013). The effectiveness of mobile-health technology-based health behaviour change or disease management interventions for health care consumers: a systematic review. PLoS Med.

[17] Whittaker R, McRobbie H, Bullen C, Rodgers A, Gu Y (2016). Mobile phone-based interventions for smoking cessation. Cochrane Database Syst Rev.

[18] Free C, Knight R, Robertson S, Whittaker R, Edwards P, Zhou W, Rodgers A, Cairns J, Kenward MG, Roberts I (2011). Smoking cessation support delivered via mobile phone text messaging (txt2stop): a single-blind, randomised trial. Lancet.

[19] Abroms LC, Whittaker R, Free C, Mendel Van Alstyne J, Schindler-Ruwisch JM (2015). Developing and pretesting a text messaging program for health behavior change: recommended steps. JMIR Mhealth Uhealth.

[20] Rodgers A, Corbett T, Bramley D, Riddell T, Wills M, Lin RB, Jones M (2005). Do u smoke after txt? Results of a randomised trial of smoking cessation using mobile phone text messaging. Tob Control.

[21] Bock B, Heron K, Jennings E, Morrow K, Cobb V, Magee J, Fava J, Deutsch C, Foster R (2013). A text message delivered smoking cessation intervention: the initial trial of TXT-2-Quit: randomized controlled trial. JMIR Mhealth Uhealth.

[22] Ybarra M, Holtrop J, Prescott T, Rahbar M, Strong D (2013). Pilot RCT results of stop my smoking USA: a text messaging-based smoking cessation program for young adults. Nicotine Tob Res.

[23] Brown KG, Gerhardt MW (2002). Formative evaluation: an integrative practice model and case study. Pers Psychol.

[24] Lee A, Sandvei M, Asmussen HC, Skougaard M, Macdonald J, Zavada J, Bliddal H, Taylor PC, Gudbergsen H (2018). The development of complex digital health solutions: formative evaluation combining different methodologies. JMIR Res Protoc.

[25] Fitts Willoughby J, Furberg R (2015). Underdeveloped or underreported? Coverage of pretesting practices and recommendations for design of text message-based health behavior change interventions. J Health Commun.

[26] Michie S, Hyder N, Walia A, West R (2011). Development of a taxonomy of behaviour change techniques used in individual behavioural support for smoking cessation. Addict Behav.

[27] Michie S, Richardson M, Johnston M, Abraham C, Francis J, Hardeman W, Eccles MP, Cane J, Wood CE (2013). The behavior change technique taxonomy (v1) of 93 hierarchically clustered techniques: building an international consensus for the reporting of behavior change interventions. Ann Behav Med.

[28] Sluta-Röka-Linjen.

[29] West R, Hajek P, Stead L, Stapleton J (2005). Outcome criteria in smoking cessation trials: proposal for a common standard. Addiction.

[30] SRNT Subcommittee on Biochemical Verification (2002). Biochemical verification of tobacco use and cessation. Nicotine Tob Res.

[31] Whittaker R, McRobbie H, Bullen C, Rodgers A, Gu Y, Dobson R (2019). Mobile phone text messaging and app-based interventions for smoking cessation. Cochrane Database Syst Rev.

[32] White IR, Royston P, Wood AM (2011). Multiple imputation using chained equations: issues and guidance for practice. Stat Med.

[33] Bendtsen M (2018). A gentle introduction to the comparison between null hypothesis testing and Bayesian analysis: reanalysis of two randomized controlled trials. J Med Internet Res.

[34] Amrhein V, Greenland S (2018). Remove, rather than redefine, statistical significance. Nat Hum Behav.

[35] Nuzzo R (2014). Scientific method: statistical errors. Nature.

[36] Wasserstein RL, Lazar NA (2016). The ASA Statement on P-values: context, process, and purpose. Am Stat.

[37] Bendtsen M (2019). Electronic screening for alcohol use and brief intervention by email for university students: reanalysis of findings from a randomized controlled trial using a Bayesian framework. J Med Internet Res.

[38] Bendtsen M (2019). An electronic screening and brief intervention for hazardous and harmful drinking among Swedish university students: reanalysis of findings from a randomized controlled trial using a Bayesian framework. J Med Internet Res.

[39] Borland R, Balmford J, Benda P (2013). Population-level effects of automated smoking cessation help programs: a randomized controlled trial. Addiction.

[40] Baker TB, Smith SS, Bolt DM, Loh W, Mermelstein R, Fiore MC, Piper ME, Collins LM (2017). Implementing clinical research using factorial designs: a primer. Behav Ther.

[41] Montgomery AA, Peters TJ, Little P (2003). Design, analysis and presentation of factorial randomised controlled trials. BMC Med Res Methodol.

[42] Gsponer T, Gerber F, Bornkamp B, Ohlssen D, Vandemeulebroecke M, Schmidli H (2014). A practical guide to Bayesian group sequential designs. Pharm Stat.

[43] Berry DA (2006). Bayesian clinical trials. Nat Rev Drug Discov.

[44] Bendtsen M (2020). The *P* value line dance: when does the music stop?. J Med Internet Res.

[45] Forouzanfar MH, Alexander L, Anderson HR, Bachman VF, Biryukov S, Brauer M, Burnett R, Casey D, Coates MM, Cohen A, Delwiche K, Estep K, Frostad JJ, Astha KC, Kyu HH, Moradi-Lakeh M, Ng M, Slepak EL, Thomas BA, Wagner J, Aasvang GM, Abbafati C, Abbasoglu Ozgoren A, Abd-Allah F, Abera SF, Aboyans V, Abraham B, Abraham JP, Abubakar I, Abu-Rmeileh NME, Aburto TC, Achoki T, Adelekan A, Adofo K, Adou AK, Adsuar JC, Afshin A, Agardh EE, Al Khabouri MJ, Al Lami FH, Alam SS, Alasfoor D, Albittar MI, Alegretti MA, Aleman AV, Alemu ZA, Alfonso-Cristancho R, Alhabib S, Ali R, Ali MK, Alla F, Allebeck P, Allen PJ, Alsharif U, Alvarez E, Alvis-Guzman N, Amankwaa AA, Amare AT, Ameh EA, Ameli O, Amini H, Ammar W, Anderson BO, Antonio CAT, Anwari P, Argeseanu Cunningham S, Arnlöv J, Arsenijevic VSA, Artaman A, Asghar RJ, Assadi R, Atkins LS, Atkinson C, Avila MA, Awuah B, Badawi A, Bahit MC, Bakfalouni T, Balakrishnan K, Balalla S, Balu RK, Banerjee A, Barber RM, Barker-Collo SL, Barquera S, Barregard L, Barrero LH, Barrientos-Gutierrez T, Basto-Abreu AC, Basu A, Basu S, Basulaiman MO, Batis Ruvalcaba C, Beardsley J, Bedi N, Bekele T, Bell ML, Benjet C, Bennett DA, Benzian H, Bernabé E, Beyene TJ, Bhala N, Bhalla A, Bhutta ZA, Bikbov B, Bin Abdulhak AA, Blore JD, Blyth FM, Bohensky MA, Bora Başara B, Borges G, Bornstein NM, Bose D, Boufous S, Bourne RR, Brainin M, Brazinova A, Breitborde NJ, Brenner H, Briggs ADM, Broday DM, Brooks PM, Bruce NG, Brugha TS, Brunekreef B, Buchbinder R, Bui LN, Bukhman G, Bulloch AG, Burch M, Burney PGJ, Campos-Nonato IR, Campuzano JC, Cantoral AJ, Caravanos J, Cárdenas R, Cardis E, Carpenter DO, Caso V, Castañeda-Orjuela CA, Castro RE, Catalá-López F, Cavalleri F, Çavlin A, Chadha VK, Chang JC, Charlson FJ, Chen H, Chen W, Chen Z, Chiang PP, Chimed-Ochir O, Chowdhury R, Christophi CA, Chuang TW, Chugh SS, Cirillo M, Claßen TKD, Colistro V, Colomar M, Colquhoun SM, Contreras AG, Cooper C, Cooperrider K, Cooper LT, Coresh J, Courville KJ, Criqui MH, Cuevas-Nasu L, Damsere-Derry J, Danawi H, Dandona L, Dandona R, Dargan PI, Davis A, Davitoiu DV, Dayama A, de Castro EF, De la Cruz-Góngora V, De Leo D, de Lima G, Degenhardt L, del Pozo-Cruz B, Dellavalle RP, Deribe K, Derrett S, Des Jarlais DC, Dessalegn M, deVeber GA, Devries KM, Dharmaratne SD, Dherani MK, Dicker D, Ding EL, Dokova K, Dorsey ER, Driscoll TR, Duan L, Durrani AM, Ebel BE, Ellenbogen RG, Elshrek YM, Endres M, Ermakov SP, Erskine HE, Eshrati B, Esteghamati A, Fahimi S, Faraon EJA, Farzadfar F, Fay DFJ, Feigin VL, Feigl AB, Fereshtehnejad SM, Ferrari AJ, Ferri CP, Flaxman AD, Fleming TD, Foigt N, Foreman KJ, Paleo UF, Franklin RC, Gabbe B, Gaffikin L, Gakidou E, Gamkrelidze A, Gankpé FG, Gansevoort RT, García-Guerra FA, Gasana E, Geleijnse JM, Gessner BD, Gething P, Gibney KB, Gillum RF, Ginawi IAM, Giroud M, Giussani G, Goenka S, Goginashvili K, Gomez Dantes H, Gona P, Gonzalez de Cosio T, González-Castell D, Gotay CC, Goto A, Gouda HN, Guerrant RL, Gugnani HC, Guillemin F, Gunnell D, Gupta R, Gupta R, Gutiérrez RA, Hafezi-Nejad N, Hagan H, Hagstromer M, Halasa YA, Hamadeh RR, Hammami M, Hankey GJ, Hao Y, Harb HL, Haregu TN, Haro JM, Havmoeller R, Hay SI, Hedayati MT, Heredia-Pi IB, Hernandez L, Heuton KR, Heydarpour P, Hijar M, Hoek HW, Hoffman HJ, Hornberger JC, Hosgood HD, Hoy DG, Hsairi M, Hu G, Hu H, Huang C, Huang JJ, Hubbell BJ, Huiart L, Husseini A, Iannarone ML, Iburg KM, Idrisov BT, Ikeda N, Innos K, Inoue M, Islami F, Ismayilova S, Jacobsen KH, Jansen HA, Jarvis DL, Jassal SK, Jauregui A, Jayaraman S, Jeemon P, Jensen PN, Jha V, Jiang F, Jiang G, Jiang Y, Jonas JB, Juel K, Kan H, Kany Roseline SS, Karam NE, Karch A, Karema CK, Karthikeyan G, Kaul A, Kawakami N, Kazi DS, Kemp AH, Kengne AP, Keren A, Khader YS, Khalifa SEAH, Khan EA, Khang YH, Khatibzadeh S, Khonelidze I, Kieling C, Kim D, Kim S, Kim Y, Kimokoti RW, Kinfu Y, Kinge JM, Kissela BM, Kivipelto M, Knibbs LD, Knudsen AK, Kokubo Y, Kose MR, Kosen S, Kraemer A, Kravchenko M, Krishnaswami S, Kromhout H, Ku T, Kuate Defo B, Kucuk Bicer B, Kuipers EJ, Kulkarni C, Kulkarni VS, Kumar GA, Kwan GF, Lai T, Lakshmana Balaji A, Lalloo R, Lallukka T, Lam H, Lan Q, Lansingh VC, Larson HJ, Larsson A, Laryea DO, Lavados PM, Lawrynowicz AE, Leasher JL, Lee JT, Leigh J, Leung R, Levi M, Li Y, Li Y, Liang J, Liang X, Lim SS, Lindsay MP, Lipshultz SE, Liu S, Liu Y, Lloyd BK, Logroscino G, London SJ, Lopez N, Lortet-Tieulent J, Lotufo PA, Lozano R, Lunevicius R, Ma J, Ma S, Machado VMP, MacIntyre MF, Magis-Rodriguez C, Mahdi AA, Majdan M, Malekzadeh R, Mangalam S, Mapoma CC, Marape M, Marcenes W, Margolis DJ, Margono C, Marks GB, Martin RV, Marzan MB, Mashal MT, Masiye F, Mason-Jones AJ, Matsushita K, Matzopoulos R, Mayosi BM, Mazorodze TT, McKay AC, McKee M, McLain A, Meaney PA, Medina C, Mehndiratta MM, Mejia-Rodriguez F, Mekonnen W, Melaku YA, Meltzer M, Memish ZA, Mendoza W, Mensah GA, Meretoja A, Mhimbira FA, Micha R, Miller TR, Mills EJ, Misganaw A, Mishra S, Mohamed Ibrahim N, Mohammad KA, Mokdad AH, Mola GL, Monasta L, Montañez Hernandez JC, Montico M, Moore AR, Morawska L, Mori R, Moschandreas J, Moturi WN, Mozaffarian D, Mueller UO, Mukaigawara M, Mullany EC, Murthy KS, Naghavi M, Nahas Z, Naheed A, Naidoo KS, Naldi L, Nand D, Nangia V, Narayan KMV, Nash D, Neal B, Nejjari C, Neupane SP, Newton CR, Ngalesoni FN, Ngirabega JdD, Nguyen G, Nguyen NT, Nieuwenhuijsen MJ, Nisar MI, Nogueira JR, Nolla JM, Nolte S, Norheim OF, Norman RE, Norrving B, Nyakarahuka L, Oh IH, Ohkubo T, Olusanya BO, Omer SB, Opio JN, Orozco R, Pagcatipunan RS, Pain AW, Pandian JD, Panelo CIA, Papachristou C, Park EK, Parry CD, Paternina Caicedo AJ, Patten SB, Paul VK, Pavlin BI, Pearce N, GBD 2013 Risk Factors Collaborators (2015). Global, regional, and national comparative risk assessment of 79 behavioural, environmental and occupational, and metabolic risks or clusters of risks in 188 countries, 1990-2013: a systematic analysis for the Global Burden of Disease Study 2013. Lancet.

[46] Bendtsen M (2020). Heterogeneous treatment effects of a text messaging smoking cessation intervention among university students. PLoS One.

[47] Badawy SM, Kuhns LM (2017). Texting and mobile phone app interventions for improving adherence to preventive behavior in adolescents: a systematic review. JMIR Mhealth Uhealth.

[48] Bendtsen M, McCambridge J (2021). Causal models accounted for research participation effects when estimating effects in a behavioral intervention trial. J Clin Epidemiol.

